# The impact of high-quality dietary patterns on the prevention of osteoporosis: a meta-analysis of observational studies

**DOI:** 10.3389/fnut.2025.1609442

**Published:** 2025-10-09

**Authors:** Jing Sui, Hanlin Yin, Linjie Zhang, Jiayi Li

**Affiliations:** ^1^Research Institute for Environment and Health, Nanjing University of Information Science and Technology, Nanjing, Jiangsu, China; ^2^Key Laboratory of Environmental Medicine Engineering, Ministry of Education, School of Public Health, Southeast University, Nanjing, China; ^3^Department of Spine Surgery, Zhuhai People’s Hospital, Zhuhai, China

**Keywords:** osteoporosis, dietary patterns, prevention, meta-analysis, bone health

## Abstract

Current prevention and treatment strategies for osteoporosis face limitations such as uncertain long-term efficacy, potential safety concerns, and poor adherence. Given these challenges, dietary interventions have emerged as a possible alternative. This study conducts a meta-analysis to systematically evaluate the association between high-quality dietary patterns and osteoporosis risk. We conducted a systematic search of PubMed and Web of Science databases through March 2025. We included observational studies that examined the association between high-quality dietary patterns (HEI, DASH, AHEI, hPDI, MeDS) and osteoporosis. The study selection followed predefined inclusion and exclusion criteria, and the Newcastle-Ottawa Scale was used for quality assessment. A total of 9 articles (including 22 studies) with 243,846 participants were ultimately included. Random-effects model analysis showed that high-quality dietary patterns overall had significant protective effects against osteoporosis (pooled OR = 0.82, 95% CI: 0.72–0.94). Subgroup analyses indicated: DASH (OR = 0.71, 95% CI: 0.57–0.90) and HEI (OR = 0.46, 95% CI: 0.33–0.66) showed significant protective effects. North America (OR = 0.85,95% CI: 0.74–0.97) and Asia (OR = 0.63,95% CI: 0.55–0.72) demonstrated protective effects. A potential protective effect (OR = 0.80,95% CI: 0.70–0.92) was shown in cross-sectional studies. The protective effect was more significant in women (OR = 0.63,95% CI: 0.53–0.74). High-quality dietary patterns, particularly DASH and HEI, may significantly reduce osteoporosis risk. Despite high heterogeneity observed in our study, results from subgroup analyses and meta-regression also supported the integration of dietary pattern into osteoporosis prevention. More cohort studies are warranted to remedy the existing limitation of inadequate longitudinal data, and additional cohort investigations are further essential for validating the observed associations between high-quality dietary patterns and osteoporosis.

**Systematic review registration:**

https://www.crd.york.ac.uk/PROSPERO/view/CRD420251009978.

## 1 Introduction

Osteoporosis, as a systemic skeletal disorder, exhibits three prominent epidemiological trends. First, the disease maintains persistently high global prevalence rates, with a meta-analysis of 343,704 participants revealing a 19.7% worldwide prevalence among adults, which is significantly higher in developing countries than in developed nations (1). Second, it directly contributes to increased bone fragility and elevated fracture risk (2). Concurrently, the age range for osteoporosis onset has expanded, and the global annual incidence of hip fractures has shown a steady upward trend (3, 4). This has drawn high attention from the public health sector.

Effective management of osteoporosis involves appropriate pharmacological interventions coupled with dietary modifications and lifestyle adjustments (5). Current clinical evidence demonstrates that although novel pharmacological interventions typified by teriparatide significantly expand therapeutic armamentarium, their long-term safety profiles and cost-effectiveness in routine clinical practice warrant comprehensive evaluation through prospective studies (5, 6). Concurrently, the clinical value of conventional calcium and vitamin D supplementation is being reassessed, while multiple systematic reviews indicate their uncertain efficacy in fracture prevention (7–9). Furthermore, although exercise therapy has been incorporated into clinical guidelines, its practical effectiveness is limited by factors including individual variability and long-term adherence issues (10, 11).

Given the limitations of current prevention and treatment strategies, exploring more effective and sustainable intervention approaches has become particularly crucial. Recent studies indicate that scientifically designed dietary pattern interventions may offer breakthroughs for osteoporosis prevention and management (12). The successful application of this intervention model has been well-documented in other chronic diseases: In cardiovascular diseases, multiple randomized controlled trials have demonstrated that the Mediterranean diet significantly reduces the risk of major cardiovascular events (13–15). For metabolic disorders, DASH diet have shown efficacy in improving glucose and lipid metabolism (16, 17). Regarding respiratory diseases, specific dietary patterns are strongly associated with improved pulmonary function and delayed disease progression (18–20). Furthermore, extensive cohort studies have revealed significant associations between healthy dietary patterns and reduced risks of various cancers (21–23).

Substantial research evidence demonstrates that dietary intervention models based on holistic dietary patterns exert positive effects on skeletal health through multi-target mechanisms. Multiple studies have confirmed significant associations between various dietary patterns and bone health indicators: a meta-analysis revealed that the Mediterranean diet was closely associated with improved bone mineral density (24), while a systematic review demonstrated its significant protective effect against fracture risk (25). The AHEI (Alternative Healthy Eating Index) dietary pattern has also been shown to prevent hip fractures (26). Research further indicates that adherence to healthy dietary patterns effectively reduces the risk of low bone mineral density (27), with even more pronounced improvements in bone mineral density and content when combined with physical activity (28). These findings collectively suggest that comprehensive dietary intervention strategies not only demonstrate high feasibility in implementation but also promote skeletal health through multi-pathway regulatory mechanisms, providing both scientific evidence and practical guidance for bone health management across all age groups.

In the present study, we conducted a comprehensive analysis of five internationally recognized high-quality dietary patterns, Healthy Eating Index (HEI), Dietary Approaches to Stop Hypertension (DASH), Alternative Healthy Eating Index (AHEI), healthy Plant-Based Diet Index (hPDI), and Mediterranean Diet (MeDS), through systematic searches of PubMed and Web of Science databases (up to March 2025). Our study investigated the association between high-quality dietary patterns and osteoporosis risk with meta-analysis, aiming to provide scientific evidence for developing dietary pattern-based prevention and management strategies for osteoporosis.

## 2 Materials and methods

The design and reporting of this meta-analysis were conducted in accordance with the Preferred Reporting Items for Systematic Reviews and Meta-Analyses (PRISMA) (29).

### 2.1 Data sources and searches

We systematically searched the online databases of PubMed and Web of Science for articles published before March 2025 on the impact of high-quality dietary patterns (HEI, DASH, AHEI, hPDI, and MeDS) on osteoporosis. We conducted a systematic literature search using the following key terms: (1) high-quality dietary pattern terms including “healthy eating Index,” “HEI,” “dietary therapy to end Hypertension,” “Dietary Approaches to Stop Hypertension Diet,” “DASH Diet,” “DASH Diets,” “Diet, DASH,” “Diets, DASH,” “Alternative healthy Eating Index,” “AHEI,” “Healthful Plant-based Diet Index,” “hPDI,” “Mediterranean Diet Score,” “Mediterranean diet,” “Mediterranean Diet,” and “MeDS”; (2) osteoporosis-related terms for Web of Science comprising “osteoporosis,” “Osteoporoses,” “Bone Loss,” “Bone Losses,” “OP,” “POP,” “PMOP,” “PMO,” “SOP,” “Osteoporotic,” “OPF,” “Osteopenia,” “Osteoblast,” “Osteoclast,” and “bone”; (3) PubMed-specific osteoporosis terms: “Osteoporoses,” “Age-Related Osteoporosis,” “Age-Related Osteoporoses,” “Age Related Osteoporosis,” “Age-Related Bone Loss,” “Age-Related Bone Losses,” “Senile Osteoporoses,” “Senile Osteoporosis,” “Post-Traumatic Osteoporoses,” and “Post-Traumatic Osteoporosis” Only publications in the English language were included in the analysis. We also referred to the reference list of the original documents to determine other relevant data.

### 2.2 Study selection and eligibility

The inclusion criteria in this study were as follows: (1) cohort studies or cross-sectional studies; (2) information about the impact of at least one of the following groups: HEI, DASH, AHEI, hPDI, and MeDS on osteoporosis; (3) directly reported hazard ratio (HR), risk ratio (RR), or odds ratio (OR) with a 95% confidence interval (CI); (4) HEI, DASH, AHEI, hPDI, and MeDS as exposure variables and the risk of osteoporosis as the outcome variable. If more than one dataset was published in different articles, we selected the latest version for analysis. During the article screening process, we excluded the following: (1) letters, reviews, case reports, or comments; (2) duplicate studies retrieved from various databases; (3) studies related to animals and cells; (4) studies that did not contain HR, RR, or OR.

We included both cohort and cross-sectional studies to leverage their complementary methodological strengths. Cross-sectional studies provide broad population representativeness via efficient data collection and large sample sizes. In contrast, cohort studies yield time-sequential exposure-outcome data, facilitating preliminary investigation of temporal relationships between dietary intake and osteoporosis onset, thereby deepening association analysis. Combining these designs was intended to expand the total sample size to boost statistical power for detecting small yet clinically meaningful associations and cross-validate findings across methodological frameworks, ultimately strengthening the meta-analysis’ robustness.

### 2.3 Data extraction

The extracted information independently identified by Jing Sui and Hanlin Yin, included type of osteoporosis, year of publication, name of the first author, research location, type of study design, research duration, age and gender of participants, sample size, type of high-quality dietary index, confounding variables adjusted for or matched in the analysis, and the OR, HR, or RR of high-quality dietary patterns and osteoporosis, as well as the corresponding 95% CI.

### 2.4 Literature quality assessment

We estimated the quality of eligible studies with the Newcastle–Ottawa Scale (NOS). We defined low-quality or high-quality studies according to the average score. The literature with a score higher than the average score was defined as high-quality.

### 2.5 Statistical analysis

This study employed OR, RR, or HR as effect measures. Between-study heterogeneity was assessed through Cochran’s Q statistic and the *I^2^* index, where an *I^2^* value of 50% or less indicated acceptable heterogeneity and warranted the use of a fixed-effect model, while an *I^2^* value exceeding 50% suggested substantial heterogeneity and justified the application of a random-effects model. When significant heterogeneity was observed, we conducted meta-regression to identify potential sources, complemented by subgroup analyses stratified by participant sex, sample size, geographic region, study design, and diet quality index type. Sensitivity analyses were performed to verify the robustness. Publication bias was assessed using Begg’s funnel plot and Egger’s linear regression test. All analyses were performed using Stata 16.0.

## 3 Results

### 3.1 Eligible studies

**Figure 1:**
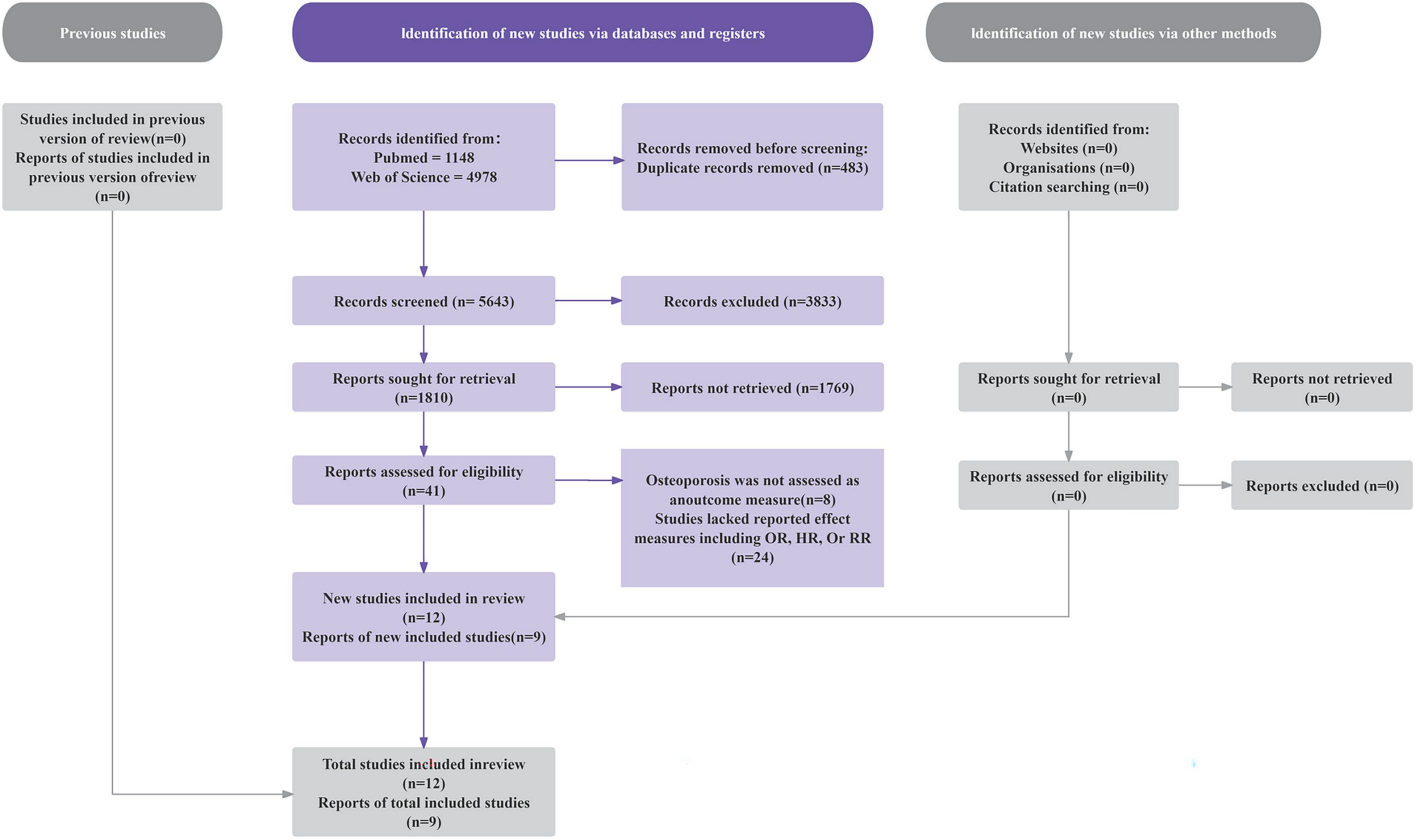
Flow diagram of the study selection. HR, hazard ratio; RR, risk ratio; OR, odds ratio.

As shown in Figure 1, our initial literature search identified 6,126 studies. After excluding 483 duplicate records, we screened 5,643 studies. We then excluded 3,520 articles involving animal or cellular research. After applying the inclusion and exclusion criteria through title and abstract review, we further eliminated 2,082 ineligible studies. Full-text assessment of the remaining 41 articles led to the exclusion of 32 studies for these reasons: (1) osteoporosis was not assessed as an outcome measure (n = 8); (2) studies lacked reported effect measures including OR, HR, or RR (n = 24). The final analysis included nine eligible articles, consisting of one prospective cohort study and eight cross-sectional studies (28, 30–37).

### 3.2 Characteristics of studies included in the meta-analysis

The characteristics of the included studies are presented in Table 1. This meta-analysis incorporated 9 studies with a total of 243,846 participants, including one prospective cohort study and 8 cross-sectional studies. The age range exceeded 12 years, with sample sizes ranging from 151 to over 200,000. All studies adjusted for major confounding factors, including age, body mass index (BMI), smoking status, physical activity, serum vitamin D levels, alcohol consumption, gender, race/ethnicity, educational attainment, and hypertension. Study quality assessed by the Newcastle–Ottawa Scale (NOS) yielded scores ranging from 6 to 8 points (mean score: 7.22).

**Table 1:** General characteristics of the included studies.

Studies	Type of index	Geographic region	Study design	Gender	Age distribution	Osteoporosis diagnostic criteria	Sample size	Variable	Nos
Li et al. (2022) (28)	DASH, AHEI, MeDS	North America	Cross-sectional	All	8–20	Bone mineral density and clinically significant fracture history	6,294	age, gender, BMI, race, family income, serum 25-hydroxyvitamin D, and serum cotinine	6
Shen et al. (2023) (30)	DASH	Asia	Cross-sectional	Male/postmenopausal female	≥50	Bone mineral density	324/515	age, BMI, height, hypertension, and diabetes with age at menarche and menopause added for women, smoking status, alcohol use, calcium supplement intake, vitamin D supplement intake, total energy intake, and physical activity	7
Hu et al. (2022) (31)	hPDI	Asia	Cross-sectional	All	68.7 ± 5.80	Bone mineral density	9,613	age, gender, BMI, SMI, educational level, income, physical activity level, history of smoking, and history of drinking, disease history	8
Zheng et al. (2024) (32)	hPDI	Europe	Cohort	All	56 ± 7.9	Bone mineral density and fracture history	202,063	age, sex, and ethnicity, BMI, Townsend deprivation index, education, physical activity, smoking status, alcohol consumption, energy intake, history of diabetes, history of hypertension, and history of hip or spine fracture	8
Zheng et al. (2023) (33)	hPDI	North America	Cross-sectional	All	≥20	Bone mineral density	10,681	age, sex, and ethnicity, education, marital status, PIR, BMI, smoking status, physical exercise, hypertension, T2DM, CKD, cancer, and history of fracture	6
Wang et al. (2024) (34)	HEI	North America	Cross-sectional	Postmenopausal female	≥50	Bone mineral density	3,421	demographics data, BMI status, smoking status, alcohol consumption, LTPA, total energy intake, serum calcium and vitamin D, and comorbidity	8
Fan et al. (2021) (35)	HEI	North America	Cross-sectional	All	≥40	Bone mineral density	10,033	age, sex/menopausal status, race/ethnicity, education, marital status, PIR, BMI, smoking status, status of hypertension, status of diabetes, taken prednisone or cortisone, history of fracture	8
Shahriarpour et al. (2020) (36)	DASH	Asia	Cross-sectional	Male	61.2 ± 8.2	Bone mineral density	151	age, body mass index, physical activity, age at menarche, age at menopause, parity, duration of lactation, and energy intake, sunlight exposure, smoking, supplement intake, and education	7
Noel et al. (2020) (37)	DASH, MeDS, AHEI	North America	Cross-sectional	Male/postmenopausal female	47–77	Bone mineral density	244/507	age, BMI and height, smoking status, season of bone mineral density measurement (fall, winter, spring, summer), osteoporosis medication use (yes/no), and calcium intake (mg/d), serum vitamin D status (mg/dL)	7

### 3.3 Meta-analysis estimates and publication Bias

**Figure 2:**
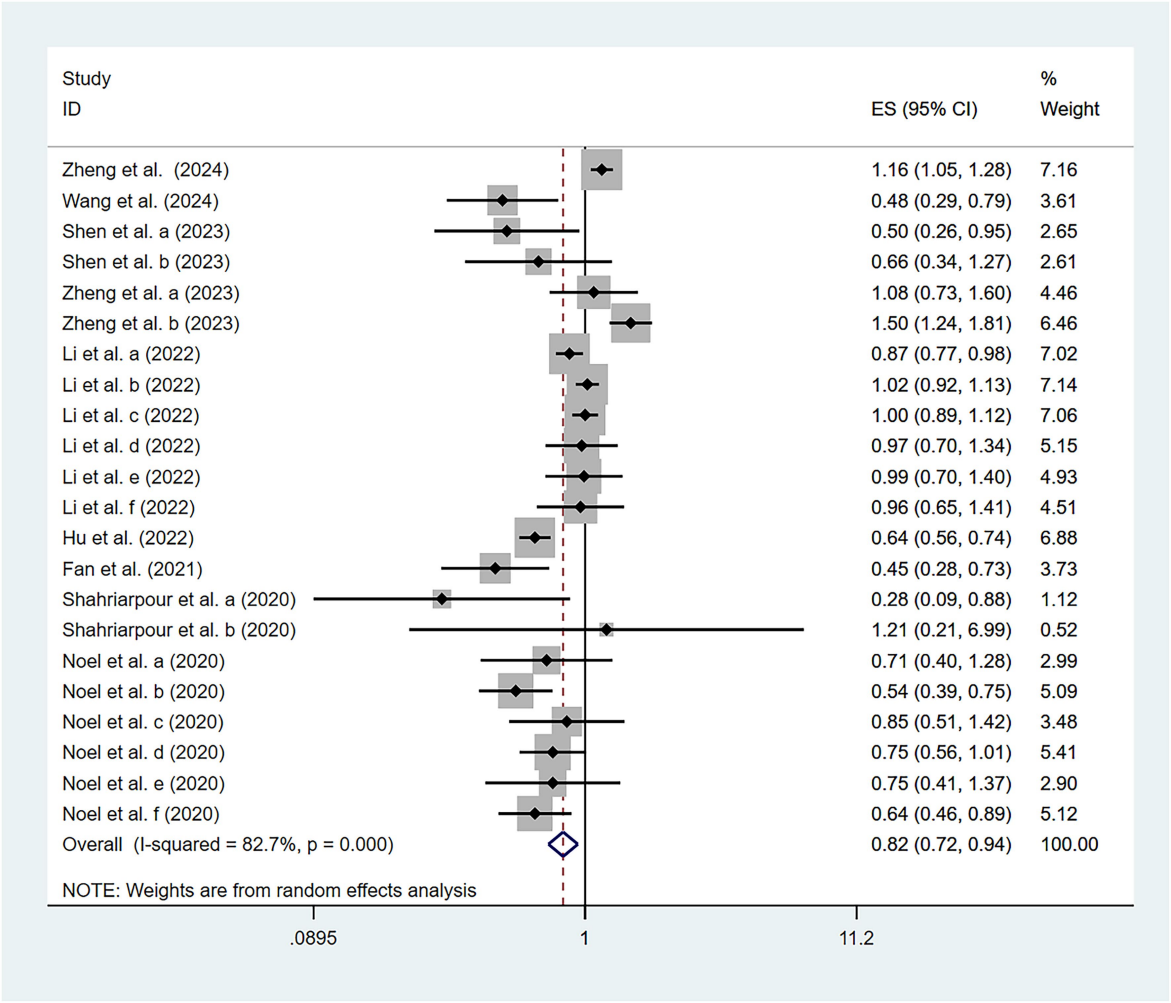
Association of high-quality dietary patterns and osteoporosis according to a random effects meta-analysis. Cl, confidence interval. Black dots: odds ratio value.

**Figure 3:**
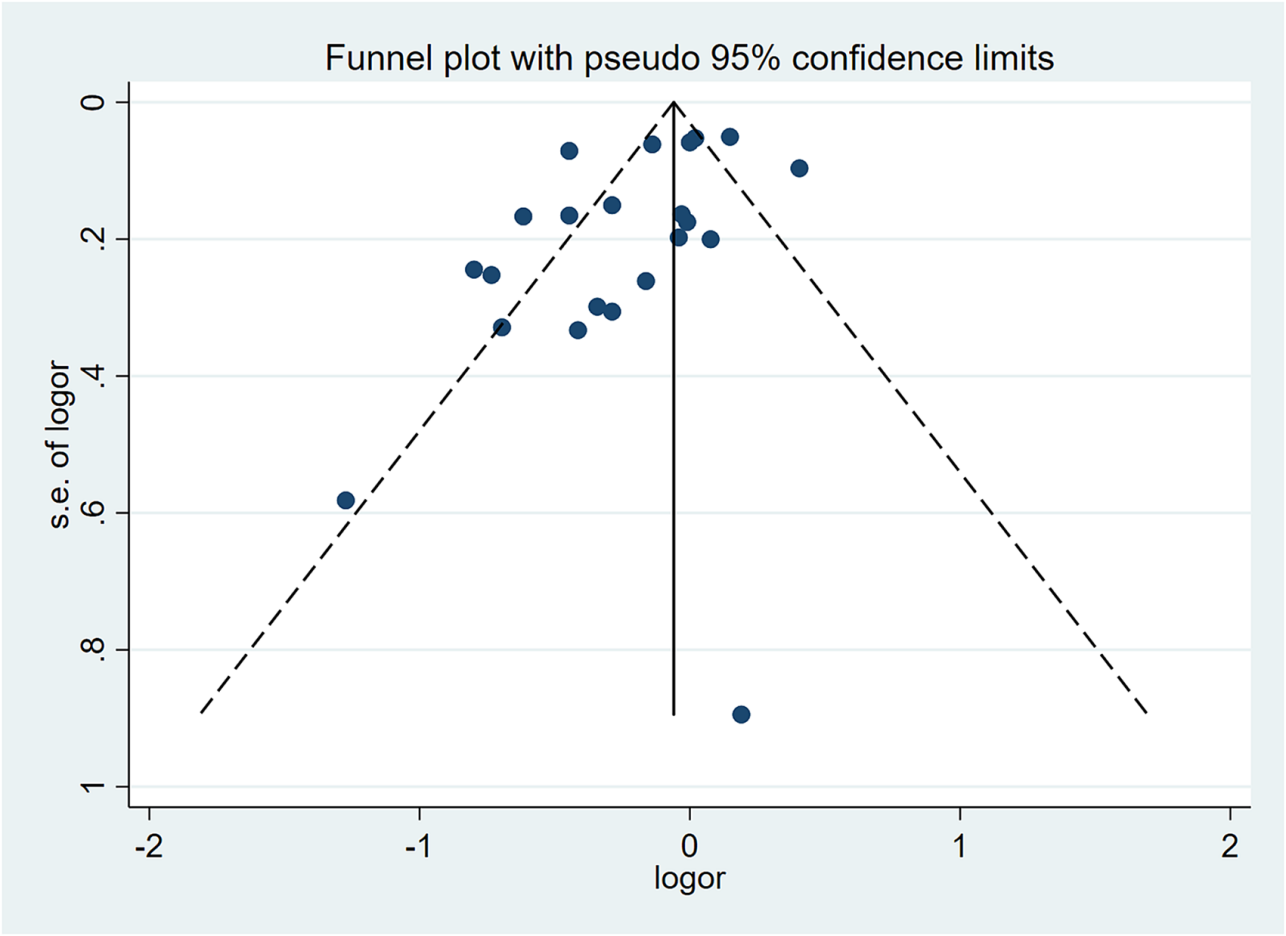
Funnel plot assessing publication bias for the association between high-quality dietary patterns and osteoporosis (random-effects model).

To examine the association between high-quality dietary patterns and osteoporosis, we performed a meta-analysis using the random effects model to pool the OR with their corresponding 95% CI. Substantial between-study heterogeneity was observed (*I^2^* = 82.7%, *p* < 0.001). To explore potential sources of heterogeneity, we conducted subgroup analyses stratified by dietary pattern type, geographic region, study design, sample size, and gender. The analysis revealed that high-quality dietary patterns exhibited a significant protective effect against osteoporosis (OR = 0.82, 95% CI: 0.72–0.94), as shown in Figure 2. Publication bias was assessed using Begg’s test (*p* = 0.159) and Egger’s test (*p* = 0.316), combined with funnel plot analysis (Figure 3). The results indicated no significant publication bias. Sensitivity analysis demonstrated consistent effect estimates upon sequential exclusion of individual studies. Specifically, the studies by Hu et al. (31), Shen et al. (30), and Zheng et al. (32) had a relatively more significant impact on the overall results. However, the differences between the results obtained after individually removing these studies and the overall results were slight. These findings confirm the robustness of our results.

Random-effects model analysis revealed significant heterogeneity among studies investigating the relationship between high-quality dietary patterns and osteoporosis. Meta-regression was performed to evaluate the influence of covariates. The results indicated that gender (*p* = 0.008), sample size (*p* = 0.027), and the type of high-quality dietary patterns (*p* = 0.010) were significant sources of heterogeneity. In contrast, study design and geographic region showed no significant effects on the odds ratios (*p* > 0.05).

### 3.4 Subgroup analysis

In the meta-regression analysis, although some covariates did not significantly influence the odds ratio, they may still affect the relationship between dietary patterns and osteoporosis in different combinations or specific contexts. To further explore these potential associations and sources of heterogeneity, we conducted subgroup analyses based on the type of high-quality dietary patterns, geographic region, study design, sample size, and gender (Table 2), aiming to comprehensively elucidate their relationship.

**Table 2:** Subgroup meta-analysis of the association between high-quality dietary patterns and osteoporosis.

Subgroup	No. of study	OR (95% CI)	*I^2^*
The type of high-quality dietary patterns			
HEI	2	0.46 (0.33,0.66)	0.0%
DASH	8	0.71 (0.57,0.90)	52.5%
AHEI	4	0.87 (0.68,1.11)	62.3%
hPDI	4	1.04 (0.71,1.54)	95.4%
MeDS	4	0.94 (0.83,1.07)	11.6%
Study region			
Europe	1	1.16 (1.05,1.28)	Na
North America	16	0.85 (0.74,0.97)	77.1%
Asia	5	0.63 (0.55,0.72)	0.0%
Study design			
Cohort	1	1.16 (1.05,1.28)	Na
Cross-sectional	21	0.80 (0.70,0.92)	80.0%
Sample size			
<1,000	13	0.75 (0.65,0.87)	27.0%
≥1,000	9	0.90 (0.76,1.08)	91.0%
Gender			
All	11	0.95 (0.82,1.11)	87.7%
Female	5	0.63 (0.53,0.74)	0.0%
Male	6	0.68 (0.52,0.90)	0.0%

The subgroup analysis revealed significant differences in the effects of various high-quality dietary patterns on osteoporosis. DASH (OR = 0.71, 95% CI: 0.57–0.90, *p* = 0.039) and HEI (OR = 0.46, 95% CI: 0.33–0.66, *p* = 0.854) demonstrated a significant protective effect against osteoporosis. In contrast, no significant associations were observed for hPDI (OR = 1.04, 95% CI: 0.71–1.54), AHEI (OR = 0.87, 95% CI: 0.68–1.11), and MeDS (OR = 0.94, 95% CI: 0.83–1.07). In terms of geographic region, high-quality dietary patterns had a significant detrimental effect on osteoporosis in Europe (OR = 1.16, 95% CI: 1.05–1.28, *p* < 0.001). In contrast, in North America (OR = 0.85, 95% CI: 0.74–0.97, *p* < 0.001) and Asia (OR = 0.63, 95% CI: 0.55–0.72, *p* < 0.001), high-quality dietary patterns showed a protective effect against osteoporosis. Regarding study design, cross-sectional studies showed a significant protective effect of high-quality dietary patterns on osteoporosis (OR = 0.80, 95% CI: 0.70–0.92, *p* < 0.001), while cohort studies indicated a detrimental effect associated with high-quality dietary patterns (OR = 1.16, 95% CI: 1.05–1.28, *p* < 0.001). This difference likely originates primarily from population specificity, as the included cohort study was limited to European participants, which may be attributed to region-specific characteristics of high-quality dietary pattern adherence and unmeasured confounding factors. When stratified by sample size (using 1,000 as the threshold), high-quality dietary patterns showed a significant protective effect against osteoporosis in small-sample studies (OR = 0.75, 95% CI: 0.65–0.87, *p* = 0.172), but no significant association was observed in large-sample studies (OR = 0.90, 95% CI: 0.76–1.08, *p* < 0.001). Finally, gender subgroup analysis revealed that high-quality dietary patterns had a significant protective effect against osteoporosis in women (OR = 0.63, 95% CI: 0.53–0.74, *p* = 0.497) and men (OR = 0.68, 95% CI: 0.52–0.90, *p* = 0.482), but no significant relationship was found in the overall population (OR = 0.95, 95% CI: 0.82–1.11, *p* < 0.001).

## 4 Discussion

With the continuous improvement of public health awareness, the relationship between dietary patterns and chronic disease prevention has received increasing scholarly attention. This study systematically analyzed nine articles (comprising data from 22 studies) involving 243,846 participants to comprehensively evaluate five internationally recognized high-quality dietary patterns (HEI, DASH, AHEI, hPDI, and MeDS) in relation to osteoporosis. The meta-analysis revealed that high-quality dietary patterns demonstrate significant protective effects against osteoporosis (OR = 0.82, 95% CI: 0.72–0.94), a finding that aligns closely with current evidence-based medical literature.

To elucidate the protective mechanisms of high-quality dietary patterns against osteoporosis, this study conducted a systematic analysis of five dietary patterns (HEI, DASH, AHEI, hPDI, and MeDS), revealing the following key shared nutritional characteristics: (1) All patterns adopt plant-based foods as dietary staples, emphasizing the fundamental role of fresh vegetables, fruits, whole grains, and legumes; (2) select vegetable oils rich in unsaturated fatty acids, especially extra virgin olive oil and cold-pressed flaxseed oil; (3) choose whole grain products such as brown rice and oats to replace refined grains; (4) incorporate nuts and seeds as key dietary elements, consuming them in proper daily portions; (5) strictly limit intake of refined sugars, processed meats, industrial trans fats, and high-sodium foods. Building upon these shared nutritional features, we propose the following potential mechanisms of action.

### 4.1 Regulation of bone turnover balance

In terms of bone turnover balance, an intervention study involving 186 adults aged 23–76 years confirmed that the DASH diet significantly reduces bone turnover (38). A systematic review showed that compared to meat-eaters, plant-based dietary patterns generally have lower intake and circulating levels of bone turnover markers (39). A study of 127 elderly men at high cardiovascular risk demonstrated that the Mediterranean diet group supplemented with virgin olive oil had significantly increased serum total osteocalcin concentrations, indicating a positive effect on promoting bone formation (40). These regulatory effects are achieved through specific bioactive components in the diet. In promoting bone formation, plant-based foods play key roles: legumes contain isoflavones that promote osteoblast differentiation by activating the Wnt/*β*-catenin pathway (41); whole grains provide magnesium that influences bone remodeling by regulating parathyroid hormone (PTH) secretion, participating in vitamin D metabolism, and modulating factors like RANKL (42); dark leafy greens rich in vitamin K1 enhance osteogenic activity through *γ*-carboxylation of osteocalcin (43, 44); extra virgin olive oil abundant in phenolic compounds can promote osteoblast activity (45). Meanwhile, dairy intake improves bone mineral density and bone metabolism markers, with particularly significant effects in populations with low calcium intake (46–48), as its rich content of protein, calcium, and vitamin D exhibits a unique “dairy matrix effect” (49). Among these, vitamin D promotes bone formation through conversion to its active form, 1,25-dihydroxyvitamin D3 (50), while proteins stimulate bone formation by increasing IGF-1 secretion (51). In terms of inhibiting bone resorption, polyphenols from olives directly act on osteoclast formation processes, reducing osteoclast numbers and thereby decreasing bone resorption extent (52); sulforaphane from cruciferous vegetables significantly inhibits osteoclast differentiation and cell fusion (53); 3,3′-diindolylmethane (DIM) can increase bone mass by inhibiting osteoclast-mediated bone resorption (54); vitamin K1 from dark leafy greens also reduces secretion of osteoclastogenic factors cytokines (44); and soy isoflavones can inhibit osteoclast precursor cell and osteoclast proliferation (55). Additionally, dietary selenium and silicon exert dual inhibitory effects by suppressing RANKL signaling and scavenging reactive oxygen species (56).

### 4.2 Enhancement of bone matrix mineralization

Regarding the maintenance of bone matrix quality, multiple clinical studies have verified it. Data from 160,106 postmenopausal women demonstrated that an anti-inflammatory diet significantly slows hip bone mineral density loss (57), while a study of 401 recruits showed that a high calcium-potassium protein diet improves tibial mineral content (58). Simultaneously, a controlled trial involving 262 postmenopausal women revealed that a healthy plant-based diet has protective effects against bone mass loss (59). This protective effect is associated with various food components: citrus fruits rich in citrus flavonoids can inhibit the synthesis and release of inflammatory cytokines, reducing inflammation-induced damage to bone matrix while decreasing reactive oxygen species production to alleviate oxidative stress damage to bone cells and maintain bone matrix quality (60). Trace elements such as zinc, copper, and magnesium from nuts and whole grains significantly enhance bone matrix mineralization efficiency by activating key pathways of osteoblast differentiation and promoting collagen synthesis (56). Dark leafy greens abundant in vitamin K1 facilitate the carboxylation of more osteocalcin to cOC, which tightly binds with hydroxyapatite in the bone matrix to maintain its structure and stability while enhancing bone toughness and strength (44). Dairy and soy products provide abundant proteins that offer structural support for bone matrix and promote bone growth and repair (51).

### 4.3 Modulation of calcium-phosphorus metabolism

The regulation of calcium-phosphorus metabolism represents another crucial target for dietary intervention. A 28-day Mediterranean diet intervention significantly increased calcium absorption and retention while reducing urinary calcium excretion and elevating bone turnover rates in adolescents (61). In a 334-participant study, the DASH diet was shown to moderately decrease calcitriol concentrations, with this effect potentially being more pronounced among African American males (62). Regarding the promotion of bone mineralization, whole grains and nuts rich in trace elements, including zinc, copper, and magnesium, significantly enhance bone matrix mineralization efficiency by activating key osteoblast differentiation pathways and stimulating collagen synthesis (56). Eggs serve as an excellent source of vitamin D that facilitates intestinal calcium transport (63). Citrus fruits, rich in vitamin C, support normal bone collagen synthesis, maintaining proper calcium uptake capacity for stable bone mass and providing a solid foundation for bone mineralization (64). Furthermore, polyphenolic compounds in olive oil may reduce arterial calcification risk by preventing abnormal calcium-phosphorus deposition (65).

### 4.4 Mitigation of inflammation and oxidative stress

The systemic regulatory effects constitute the fourth mechanism of dietary protection. The Composite Dietary Antioxidant Index (CDAI) study demonstrated that among 11,664 middle-aged and elderly individuals, the group with the highest CDAI scores showed a significantly reduced risk of osteoporosis (66). This protective effect originates from multiple components: polyphenols from berries and dark-colored fruits alleviate oxidative stress by activating the Nrf2-ARE pathway (67, 68); and butyrate produced through gut microbial fermentation of dietary fiber from whole grains and legumes can inhibit osteoclast precursor cell proliferation (69). A randomized controlled trial involving 102 women with migraines confirmed that after 8 weeks on the DASH diet, oxidative stress marker MDA levels were 27% lower compared to the control group (70). Meanwhile, a cross-sectional study of 391 obese women indicated that reduced red meat consumption led to decreased inflammatory factor levels (71).

This meta-analysis systematically evaluated the relationship between high-quality dietary patterns and osteoporosis, revealing that high-quality dietary patterns have significant protective effects against osteoporosis, though with certain heterogeneity among studies. To explore the sources of heterogeneity, we conducted multiple subgroup analyses. Through sensitivity analysis, we verified consistency among included studies and employed meta-regression models to explain potential heterogeneity, thereby enhancing the generalizability and reliability of the findings.

Subgroup analysis results showed that several high-quality dietary patterns did not demonstrate statistically significant effects on osteoporosis. For instance, hPDI (OR = 1.04, 95% CI: 0.71–1.54, *p* < 0.001), AHEI (OR = 0.87, 95% CI: 0.68–1.11, *p* = 0.047), and MeDS (OR = 0.94, 95% CI: 0.83–1.07, *p* = 0.335) did not show significant associations with osteoporosis. This outcome may be influenced by several factors. First, smaller sample sizes in certain subgroups (such as specific age groups or genders) may have reduced statistical power, making it difficult to detect significant associations. Second, different methods for creating dietary indices and inconsistent standards for assessing individual adherence to dietary patterns, for example, MeDS and DASH using population-based cutoffs (gender-specific median intake values adjusted for total energy and quintiles) while other indices (AHEI) are based on existing nutritional knowledge and dietary recommendations, led to considerable variation across studies, resulting in non-significant findings. Additionally, most study designs were cross-sectional and unable to exclude confounding factors, thereby diluting observed association strengths. Notably, since both the hPDI and MeDS diets are predominantly plant-based, research suggests potential harmful associations between plant-based diets and bone health (33), which may somewhat diminish the protective effects of high-quality dietary patterns against osteoporosis, contributing to nonsignificant results.

In the present study, meta-regression identified gender (*p* = 0.008), sample size (*p* = 0.027), and dietary pattern type (*p* = 0.010) as key sources of heterogeneity in the association between high-quality dietary patterns and osteoporosis. For gender, the more pronounced protective effects in female and male compared to the overall population were driven by sex-specific hormonal dynamics and dietary adherence patterns. Postmenopausal women experience estrogen decline that accelerates bone resorption (72), making their skeletal health more responsive to bone-protective nutrients in diets like DASH/HEI. While bone metabolism in male is sustained by testosterone (73), reducing the relative magnitude of dietary benefit. Additionally, studies found higher adherence to structured dietary patterns in female, which reinforced consistent nutrient intake (74). Regarding sample size, the small sample size of the included studied may introduce bias, potentially affecting the accuracy of the observed impact in this study (75). For dietary pattern type, HEI and DASH delivered significant protection by prioritizing bone-targeted nutrients (30, 76), whereas hPDI lacked significance partly due to its predominantly plant-based framework. It was consistent with Zheng et al. (33) suggesting potential adverse links between plant-based diets and bone health that offset protective effects. Moreover, AHEI were less specifically tailored to bone health compared to HEI/DASH. Meanwhile, the dietary pattern scoring methodologies exhibit key limitations and variability. HEI prioritizes bone-protective nutrients with nutrient-adequacy weights, while MeDS focuses on Mediterranean staples and regional traditions. These inconsistencies reduce between-study comparability, drive high meta-analysis heterogeneity (*I*^2^ = 82.7%), and confound pooled results, such as non-significant associations for hPDI and MeDS in non-Mediterranean regions. Different scoring method for dietary patterns, cultural dietary differences, dietary survey method, study area, and sample size might be the main sources of the result.

The primary strength of this meta-analysis lies in its inclusion of multiple studies covering five different high-quality dietary patterns (HEI, DASH, AHEI, hPDI, and MeDS), with a large sample size (totaling 243,846 participants) providing strong statistical power. The study employed random-effects models and further validated result robustness through sensitivity analysis and publication bias testing. Moreover, meta-regression and subgroup analyses explored heterogeneity sources, enhancing result reliability and generalizability, demonstrating that high-quality dietary patterns, particularly DASH and HEI, have significant protective effects against osteoporosis.

However, our study has several limitations. First, included studies exhibited substantial heterogeneity (*I*^2^ = 82.7%), and although adjusted through subgroup analyses and meta-regression models, some heterogeneity sources remained unexplained, such as inconsistencies in dietary assessment tools and differences in participant baseline characteristics. Second, with study designs primarily being cross-sectional, establishing causal relationships was challenging, future cohort studies are needed to further validate these findings. Meanwhile, cross-sectional and cohort studies differ in inferential capacity. Cross-sectional studies cannot establish temporal precedence between dietary patterns and osteoporosis, thereby introducing recall bias in self-reported dietary intake. Whereas the single cohort study mitigates reverse causality by measuring dietary patterns prior to osteoporosis diagnosis but has limited generalizability due to its exclusive focus on European participants. Additionally, merging these two designs has contributed to high between-study heterogeneity owing to variations in their confounding adjustment strategies. Finally, limited sample sizes for some dietary patterns (hPDI, AHEI, and MeDS) may have increased result uncertainty, necessitating larger-sample studies to clarify these patterns’ specific effects.

## 5 Conclusion

In conclusion, our findings demonstrate that high-quality dietary patterns, particularly the DASH and HEI diets, exhibit potentially protective effects against osteoporosis, whereas the effects of hPDI, AHEI, and MeDS diets require further investigation. More cohort studies are warranted to remedy the existing limitation of inadequate longitudinal data, and additional cohort investigations are further essential for validating the observed associations between high-quality dietary patterns and osteoporosis.

## Data Availability

The raw data supporting the conclusions of this article will be made available by the authors, without undue reservation.
